# Integrating PANoptosis insights to enhance breast cancer prognosis and therapeutic decision-making

**DOI:** 10.3389/fimmu.2024.1359204

**Published:** 2024-03-05

**Authors:** Shu Wang, Zhuolin Li, Jing Hou, Xukui Li, Qing Ni, Tao Wang

**Affiliations:** ^1^ Department of Breast Surgery, Guizhou Provincial People’s Hospital, Guiyang, Guizhou, China; ^2^ Medical College, Guizhou University, Guiyang, Guizhou, China; ^3^ Research Laboratory Center, Guizhou Provincial People’s Hospital, Guiyang, Guizhou, China; ^4^ NHC Key Laboratory of Pulmonary Immune-related Diseases, Guizhou Provincial People’s Hospital, Guizhou University, Guiyang, Guizhou, China

**Keywords:** breast cancer, PANoptosis, machine learning, immunotherapy, BI-2536

## Abstract

**Background:**

Despite advancements, breast cancer outcomes remain stagnant, highlighting the need for precise biomarkers in precision medicine. Traditional TNM staging is insufficient for identifying patients who will respond well to treatment.

**Methods:**

Our study involved over 6,900 breast cancer patients from 14 datasets, including in-house clinical data and single-cell data from 8 patients (37,451 cells). We integrated 10 machine learning algorithms in 55 combinations and analyzed 100 existing breast cancer signatures. IHC assays were conducted for validation, and potential immunotherapies and chemotherapies were explored.

**Results:**

We pinpointed six stable Panoptosis-related genes from multi-center cohorts, leading to a robust Panoptosis-model. This model outperformed existing clinical and molecular features in predicting recurrence and mortality risks, with high-risk patients showing worse outcomes. IHC validation from 30 patients confirmed our findings, indicating the model’s broader applicability. Additionally, the model suggested that low-risk patients benefit more from immunotherapy, while high-risk patients are sensitive to specific chemotherapies like BI-2536 and ispinesib.

**Conclusion:**

The Panoptosis-model represents a major advancement in breast cancer prognosis and treatment personalization, offering significant insights for effectively managing a wide range of breast cancer patients.

## Introduction

Breast cancer remains a significant health challenge globally, being one of the leading causes of cancer-related deaths among women (1). Despite advancements in early detection and treatment strategies aiming to reduce recurrence and mortality, the battle against breast cancer continues (2). The quest for improved prognosis and therapeutic outcomes is ongoing, with early diagnosis playing a crucial role in enhancing survival rates (3).

Recent scientific developments have shed light on PANoptosis, a sophisticated form of programmed cell death that integrates elements of pyroptosis, apoptosis, and necroptosis (4). This process is crucial for maintaining the body’s balance and defending against diseases by removing harmful cells (5). Intriguingly, PANoptosis has been implicated in the progression of various diseases, including cancer, highlighting its potential as a target for innovative treatments (6). For example, research has indicated that manipulating PANoptosis pathways could influence the development of tumors and the effectiveness of cancer therapies (7).

Despite the known significance of programmed cell death mechanisms like apoptosis in cancer, the specific contributions of PANoptosis to breast cancer progression and patient outcomes remain underexplored (8). While prognostic models based on PANoptosis-related genes have shown promise in other cancers, a dedicated model for breast cancer prognosis is yet to be established (9). This gap underscores the need for a deeper understanding of PANoptosis in breast cancer, which could unlock new avenues for diagnosis and treatment (10).

This study aims to bridge this gap by conducting a thorough analysis of breast cancer samples from the TCGA-BRCA and GEO datasets. By employing cutting-edge single-cell sequencing technologies and a comprehensive set of machine learning techniques, we have identified critical PANoptosis-related genes associated with breast cancer outcomes. Using these insights, we developed a novel prognostic model that categorizes breast cancer patients into distinct risk groups, offering a new tool for predicting survival and guiding treatment decisions. Our model’s effectiveness was rigorously tested across multiple datasets, and we further investigated the molecular and immunological profiles of the risk categories identified, providing a comprehensive view of the implications of PANoptosis in breast cancer.

## Materials and methods

### Data acquisition

The foundation of our research involved the compilation of PANoptosis genes, encompassing elements of pyroptosis, apoptosis, and necroptosis, which were meticulously curated from GeneCards, GSEA gene sets, KEGG pathways, and relevant literature (11, 12). This curation process yielded a comprehensive list of genes integral to the PANoptosis pathway, detailed in **Supplementary Table S1**.

The training dataset was assembled from the TCGA database, which included gene profiles, mutational data, and clinical information from breast cancer cases. Samples without survival data were carefully excluded from the dataset to ensure data completeness and accuracy.

To enhance the robustness of our findings and validate our model, we obtained additional datasets from the Gene Expression Omnibus (GEO) database and MetaGxData (13). These validation datasets were comprised of samples from various studies, namely GSE93601, GSE76250, GSE70947, GSE20685, GSE131769, GSE96058, GSE20711, GSE24450, GSE202203, GSE21653, GSE86166, GSE48391, GSE88770 and PNC. This comprehensive approach allowed us to validate our results across diverse datasets and strengthen the reliability of our findings.

### Single-cell analysis

Single-cell RNA sequencing data for breast cancer was sourced from the GEO database (accession number GSE161529) as the basis for our single-cell analysis (14). Our preprocessing protocol began with the elimination of genes that were not expressed in any samples, specifically those with zero counts across all cells. This step was critical to focus our analysis on active genetic elements within the samples. We then normalized the gene expression matrix. This normalization process, conducted using the “SCTransform” function within the Seurat R package, allowed for the correction of technical variances and the stabilization of variance across features. Subsequent dimensionality reduction techniques, including PCA, tSNE, and UMAP, were employed to distill the high-dimensional data into a more interpretable form, facilitating the identification of cellular phenotypes and states. Cell populations were categorized using the “FindNeighbors” and “FindClusters” functions, which are instrumental in discerning the heterogeneity within the cell populations. We augmented our quality control measures by identifying and removing doublets with the DoubletFinder R package, further ensuring the integrity of our dataset (15).

Following these rigorous quality control measures, we retained approximately 37,451 cells for subsequent analyses. The final step involved cell type assignment; a task made efficient using Celltypist (16). This comprehensive approach ensured the robust processing and analysis of the single-cell data, setting a strong foundation for our research endeavors.

### CellChat analysis

For the investigation of intercellular communication within the tumor microenvironment, we utilized the “CellChat” R package, which allows for the analysis of cell-cell interactions based on ligand-receptor pairs (17). We constructed CellChat objects for each group using their respective UMI count matrices. The “CellChatDB.human” database was employed as the reference for known ligand-receptor interactions, enabling us to analyze the complex signaling networks within our samples. Using the default settings within CellChat, we performed a comparative analysis of the interaction counts and strengths between different cell types. To synthesize this information across groups, we merged the individual CellChat objects using the “mergeCellChat” function. This step was crucial for aggregating data to observe broader trends in cell communication. Differences in interaction number and strength among specific cell types across groups were visualized using the “netVisual_diffInteraction” function. We ascertained variations in signaling pathways through the “rankNet” function and depicted the spread of signaling gene expression across groups with “netVisual_bubble” and “netVisual_aggregate” functions.

### Functional analyses

To elucidate the complex landscape of differential PANoptosis-related gene expression between tumor and normal tissues, we utilized the GO and KEGG databases for a thorough assessment of associated functional activities and pathways (18, 19). The Enrichplot package within R was employed to visualize the results of this enrichment analysis. In parallel, the clusterProfiler algorithm facilitated Gene Set Enrichment Analysis (GSEA) to distinguish the biological functions between distinct breast cancer risk subgroups identified by our model (20). We established a False Discovery Rate (FDR) below 0.05 to denote statistical significance, enhancing the robustness of our findings by performing 1,000 permutations for each analysis. This comprehensive approach allowed us to identify key functional pathways differentially activated in our PANoptosis-related gene sets, providing insight into the molecular underpinnings of breast cancer pathology and prognosis.

### Establishment of the PANoptosis score

To uncover the significance of PANoptosis in BC, a systematic methodology was adopted. This exploration commenced with a differential analysis, specifically comparing gene expression patterns between tumor and normal tissues using the TCGA-BRCA dataset.

To visually depict the outcomes of differential gene expression, we employed a heatmap, effectively illustrating the observed disparities. Concurrently, we analyzed gene correlations, utilizing the igraph package. The crucial PANoptosis Score was then meticulously calculated. This calculation was based on the differentially expressed PANoptosis-related genes. In this effort, we utilized the ssGSEA algorithm for bulk data analysis (21), while for single-cell data, we employed the Ucell algorithm (22). This dual approach ensured a comprehensive and robust assessment of the PANoptosis Score, facilitating a deeper understanding of its role in breast cancer.

### Development and validation of the PANoptosis-model

In constructing a prognostic model for breast cancer based on PANoptosis, we followed the analytical workflow established by Liu et al. (23). We integrated ten classical computational algorithms, including Random Forest (RSF), Least Absolute Shrinkage and Selection Operator (LASSO), Gradient Boosting Machine (GBM), Survival Support Vector Machine (Survival-SVM), Supervised Principal Components (SuperPC), Ridge Regression, Partial Least Squares Regression for Cox (plsRcox), CoxBoost, Stepwise Cox, and Elastic Network (Enet). each bringing unique strengths in dimensionality reduction and variable selection, as detailed in **Supplementary Table S2**. The TCGA-BRCA dataset served as the training cohort, with the combination of these algorithms being used to create the prognostic signature. We then evaluated the model’s predictive power using the average concordance index (C-index) across five external test cohorts from the GEO database. This process allowed us to identify the most effective prognostic model for breast cancer, which we refer to as the PANoptosis-model:


$$riskscore=\sum_{i=1}^{n}\left(\beta_{i}\times Exp_{i}\right)$$

Where ‘n’ represents the number of PANoptosis genes, ‘Exp’ signifies the PANoptosis gene profile, and ‘β’ denotes the multi-Cox coefficient.

This model calculates a risk score based on the expression profile of PANoptosis genes and their respective coefficients derived from multivariate Cox regression. Patients from the TCGA-BRCA dataset were stratified into different risk groups according to these scores. The generalizability of the risk score was then validated using additional external datasets, which served as independent test cohorts. Kaplan-Meier survival analysis, conducted with R v4.2, was employed to discern survival differences between the risk groups, with a p-value of less than 0.05 indicating statistical significance. This meticulous approach ensured that the PANoptosis-model was robustly validated and capable of accurately predicting patient outcomes.

### Genomic character analysis

To unravel the genomic alteration disparities between the PANoptosis-model subgroups, we conducted an extensive examination of mutation and Copy Number Alteration (CNA) data within the TCGA-BRCA dataset.

We initiated this analysis by extracting the raw mutation file and proceeded to calculate the Tumor Mutation Burden (TMB) for each sample. To provide insights into the genetic landscape, we visually represented the top 28 genes utilizing the maftools package. Following the methodology described by Wang et al. (24), we employed the deconstructSigs package to derive mutational signatures unique to each patient. Notably, we highlighted four signatures with notable occurrence frequencies in BRCA: SBS1, SBS3, SBS11, and SBS12.

Furthermore, we selected the top 5 regions exhibiting a high-level CNA frequency. Particular attention was given to genes within chromosomes 13q34, including CDK19, SOBP, ATG5, and FYN. This comprehensive analysis provided valuable insights into the genomic alterations within the PANoptosis-model subgroups.

### Estimation of TME variations

We collected five algorithms [MCPcounter (25), xCell (26), CIBERSORT (27), quanTIseq (28), and TIMER (29)] to estimate the abundance of diverse immune cells through different risk score groups using the IOBR package (30). Furthermore, we utilized ESTIMATE and TIDE to assess the composition, structure, and state of the tumor microenvironment (31, 32). This analysis provided crucial insights into the biological traits and prognosis of the tumor. Finally, the expression features of multiple immune checkpoints were also quantified to explore the immune state, preliminary predicting therapeutic sensitivity to ICIs therapy.

### Selections of therapeutic targets and agents

For estimating drug targets and predicting chemotherapeutic responses, we obtained comprehensive target data for 6,125 compounds from the Drug Repurposing Hub (https://clue.io/repurposing), resulting in 2,249 distinct drug targets after removing duplicates (33). We used Spearman correlation analysis to identify potential drug targets associated with unfavorable prognosis by correlating the gene expression of targetable genes with risk scores (correlation coefficient > 0.25, P< 0.05). Subsequently, we correlated CERES scores with risk scores for brain cell lines from CCLE, identifying genes (correlation coefficient < -0.2, P< 0.05) associated with poor prognosis dependence (34).

To predict drug responses accurately, we leveraged CTRP and PRISM datasets, which contain extensive drug screening and molecular data across cancer cell lines. We performed differential expression analyses between bulk and cell line samples. For drug response prediction, we employed the reliable ridge regression model within the pRRophetic package. This model was trained on expression profiles and drug response data from solid Cancer Cell Lines (CCLs) and exhibited robust performance, validated by default 10-fold cross-validation (35).

Additionally, we conducted a Supplementary Connectivity Map (CMap) analysis to assess the therapeutic potential of candidate agents in BC (36). We performed differential gene expression analysis between tumor and normal tissue samples and then submitted the top 300 genes (150 up-regulated and 150 down-regulated) to the dedicated CMap website (https://clue.io/query). This analysis drew on gene expression signatures from CMap v1 and the LINCS database. Negative connectivity scores indicated the potential therapeutic efficacy of perturbations in the disease context.

### Human sample collection and IHC staining

In this study, we obtained specimens from a cohort of 30 patients diagnosed with BC at the Guizhou Provincial People’s Hospital. These specimens were collected during surgical procedures. Hematoxylin and eosin (HE) staining was applied to the specimens based on established protocols. Diagnostic evaluations were independently conducted by two pathologists. Comprehensive cohort details are provided in **Supplementary Table S3**.

Immunohistochemistry (IHC) was conducted on paraffin-embedded samples, adhering to methods outlined in our earlier publications (37, 38). The antibodies utilized are enumerated in **Supplementary Table S4**. Evaluation was consistent with established protocols and scoring guidelines. Two pathologists independently assessed protein expression levels, consistent with the methodology described in our previous work (38).

### qRT-PCR and patient stratification

RNA was isolated from breast cancer samples using TRIzol reagent (Invitrogen, Carlsbad, CA, USA). This was followed by the synthesis of cDNA and qRT-PCR procedures, employing GoScript reverse transcriptase and Master Mix (Promega), following the manufacturer’s instructions. Data were captured using the CFX96 Touch Real-Time PCR Detection System (BioRad, Hercules, CA, USA). The relative quantification of gene expression was performed with the 2^-ΔΔCq^ method, normalizing against GAPDH as the reference gene.

Patient stratification into low-risk and high-risk categories was achieved through the evaluation of gene expression levels, applying a specific threshold based on the PANoptosis-model’s equation.

### Statistical analysis

Data processing and statistical analysis were conducted using R software (version 4.2.3). We applied the Wilcoxon signed-rank test to evaluate expression differences between BC patients and controls. Pearson and Spearman correlation analyses were utilized to determine statistical correlations between parametric and non-parametric variables, respectively. Significance was established at a p-value< 0.05, with gradations indicated as *p< 0.05, **p< 0.01, ***p< 0.001, ****p< 0.0001.

## Results

### Differential expression of PANoptosis genes in breast cancer tissues

**Figure 1:**
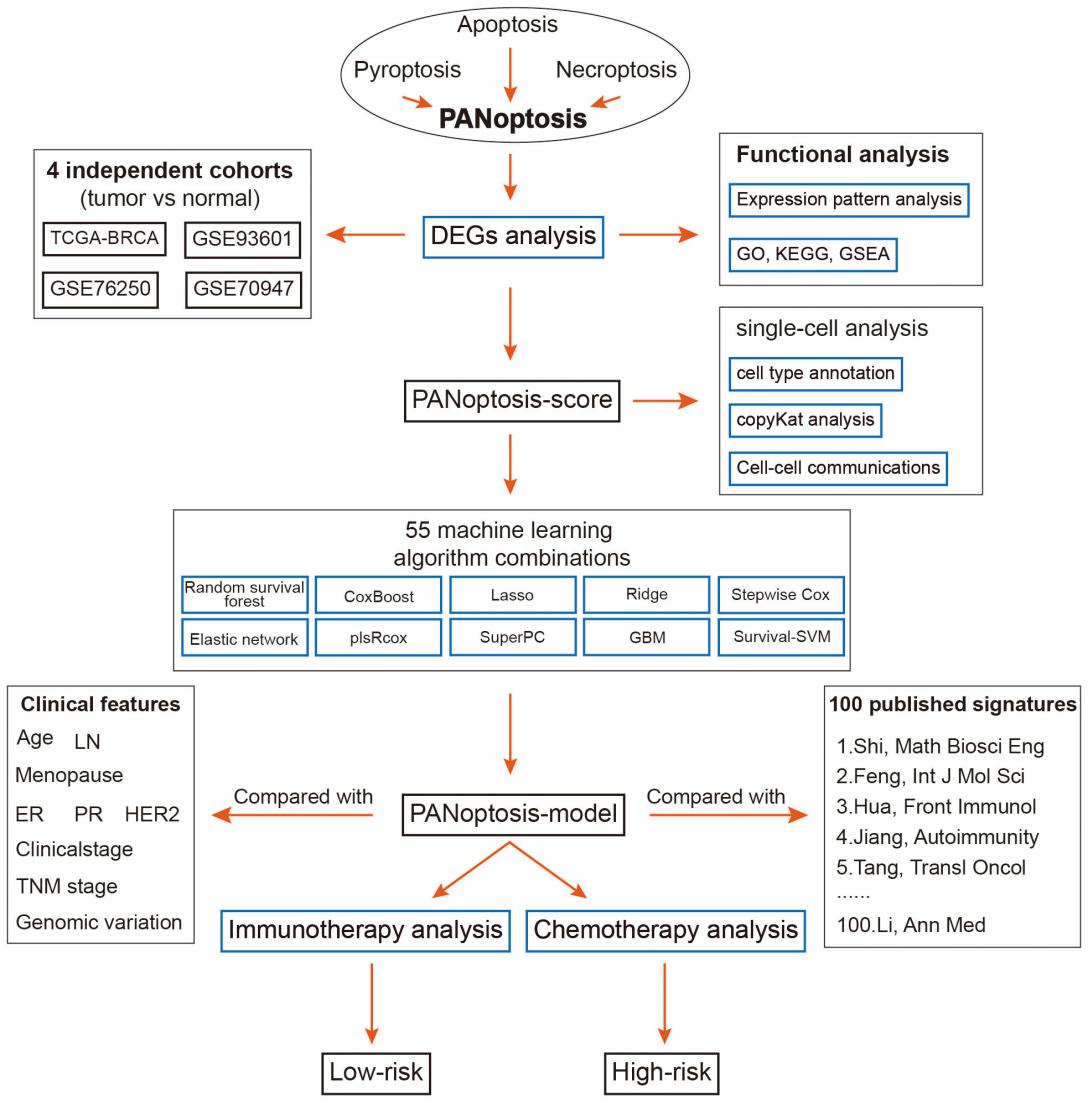
The overall flow of this study.

**Figure 2:**
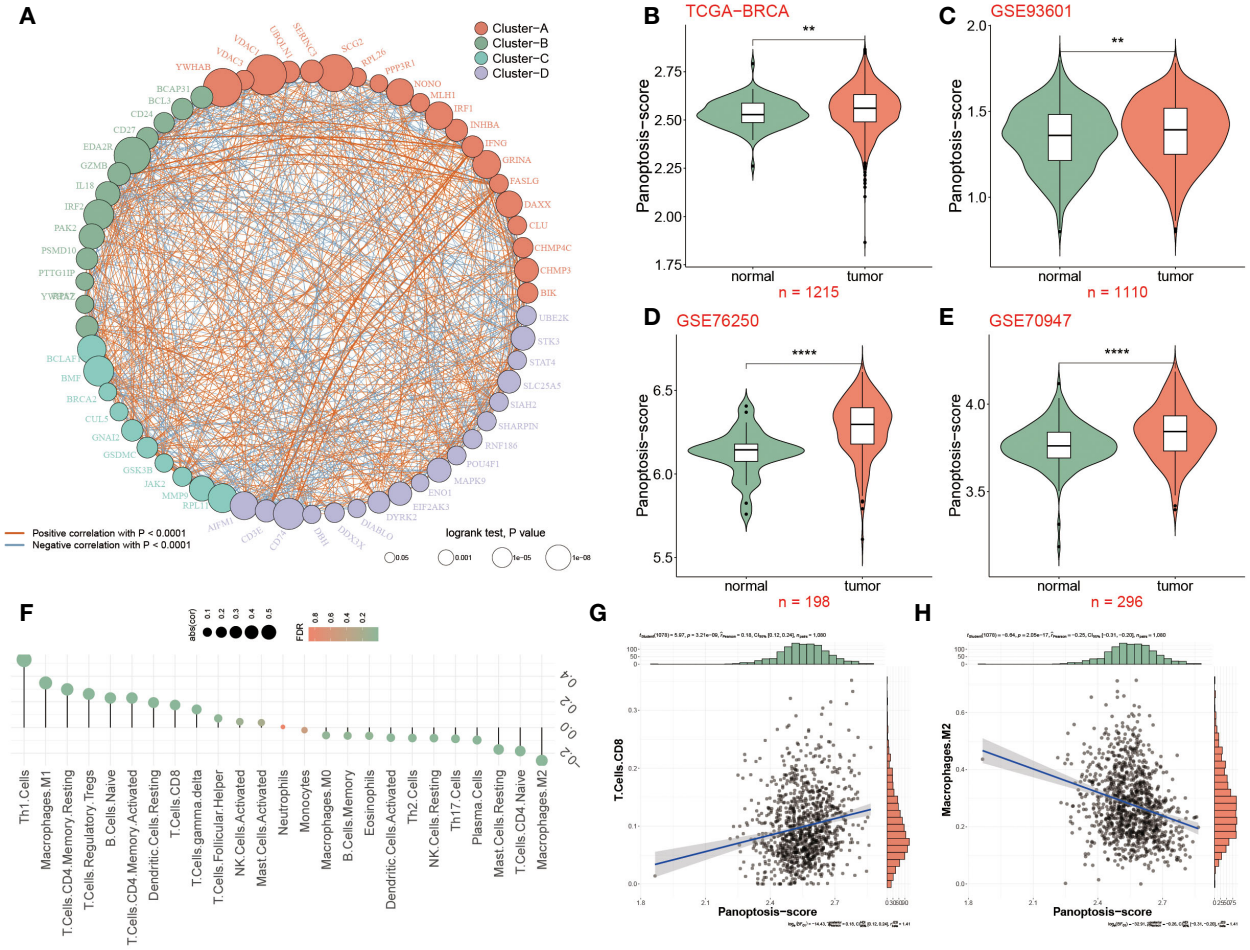
Differential expression of PANoptosis genes in breast cancer tissues. **(A)** 61 differentially expressed PANoptosis genes were categorized into four cell clusters (A, B, C, and D), and this network revealed the complicated relationships between these genes. The red or blue line represented a positive or negative correlation. The larger the circle, the stronger the impact of a single gens on BC. **(B)** The PANoptosis-score was compared among two groups in the TCGA-BRCA dataset, **(C–E)** Validation datasets of GSE93601 **(C)**, GSE76250 **(D)** and GSE70947 **(E)** for PANoptosis-score. **(F)** Relevance of PANoptosis-score and immune cell infiltration. **(G, H)** The correlation between PANoptosis-score and CD8^+^T cells and M2 macrophages. **P<0.01, ****P < 0.0001.

The overall design of this study is displayed in **Figure 1**. We identified 52 pyroptosis genes, 581 apoptosis genes, 101 necroptosis genes, and 28 potential PANoptosis genes. To comprehensively evaluate the expression landscape of PANoptosis-related genes in BC, we conducted differential gene expression analysis comparing tumor samples to normal counterparts within the TCGA-BRCA dataset. The heatmap depiction underscores a pervasive pattern of dysregulated PANoptosis gene expression within BC samples, delineating a stark contrast between malignant and non-malignant tissue profiles (**Supplementary Figure S1A**; **Supplementary Table S5**). In our subsequent analysis, we discerned 61 genes with prognostic significance within the realm of PANoptosis, segregating these into four distinct clusters of expression (as depicted in **Figure 2A**). A particularly compelling positive correlation emerged between IFNG and FASLG within cluster A (cor = 0.84, P-value< 0.001), suggesting a concerted regulatory mechanism at play. Similarly, cluster B showcased a noteworthy synergistic expression pattern between YWHAZ and CD24 (cor = 0.300, P-value< 0.001). In stark contrast, cluster D revealed an intriguing negative correlation between POU4F1 and SIAH2 (cor = -0.34, P-value< 0.001), hinting at an intricate antagonistic interaction pertinent to BC progression (**Figure 2A**).

Further refining our investigative scope, we introduced a novel metric, the PANoptosis-score, crafted to quantify the cumulative activity of PANoptosis pathways within BC. Our scrutiny extended across the foundational TCGA-BRCA dataset and was corroborated by analyses within three ancillary datasets (GSE93601, GSE76250, and GSE70947). The data cohesively pointed to an elevated PANoptosis-score in BC patients relative to normal groups, a finding consistently replicated across all datasets examined (**Figures 2B–E**). The enrichment analyses were conducted to clarify the function and pathways of these genes within BC patients. The Proteomaps indicated that these differentially expressed genes related to PANoptosis displayed strong relationships with 15 top roles, such as signal transduction, signaling molecules and interaction, and transcription. Massive signaling pathways were exhibited including IL19, IL16, PDX1 and IL33 (**Supplementary Figure S1B**).

Based on the background that tumor microenvironment (TME) participates in the progression of tumors, the association between the PANoptosis-score and 26 infiltrated immune cells was further elaborated as unraveled in **Figure 2F**, of which Th1 cells, Tregs and M1 macrophages were positively infiltrated with PANoptosis-score in BC patients, in contrast, taking M2 macrophages as an example, 11 immune cells were negatively correlated with PANoptosis-score. According to the correlation analysis results from **Figures 2G, H**, it was confirmed that the PANoptosis-score exhibited a positive relevance with CD8^+^T cells, but a negative relation to M2 macrophages.

### Single-cell analysis reveals PANoptosis dynamics in BC

**Figure 3:**
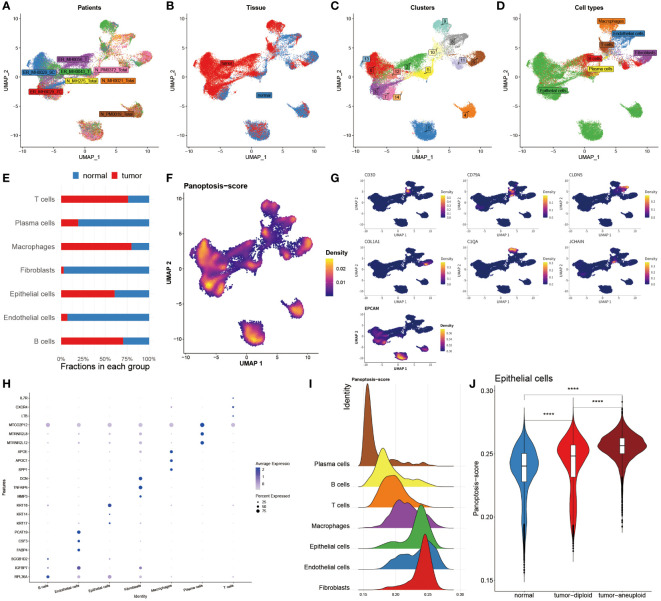
Single-cell analysis reveals PANoptosis dynamics in BC. **(A)** Distribution of cells collected from 8 patients. **(B)** Distribution of cells from tumor and normal tissues. **(C)** Distribution of 15 cell clusters. **(D)** Distribution of 7 annotated cell types. **(E)** Bar chart unveiling the proportion of 7 immune cells in two groups. **(F)** PANoptosis activity in each cell. **(G)** Specific marker genes for each immune cell. **(H)** Top three marker genes in each immune cell. **(I)** Mountain map exhibiting PANoptosis-score in each cell type. **(J)** Violin map revealing the difference of PANoptosis-score among normal tissues, tumor-diploid, and tumor-aneuploid in epithelial cells. ****P < 0.0001.

To further assess the PANoptosis features in BC, the single-cell transcriptome analysis was performed, of which a total of eight patients and two groups (tumor and normal) were enrolled in this analysis (**Figures 3A, B**). We then grouped them into fifteen cell clusters and identified seven cell types (**Figures 3C, D**). The bar chart clearly and intuitively presented the proportion of seven cell types in normal and BC tissues, of which three cell types, including T cells, macrophages, and epithelial cells, accounted for far over fifty percent in tumor tissues relative to the normal tissues, and conversely, Pericytes, fibroblasts and endothelial cells occupied higher proportion in normal tissues (**Figure 3E**). To accurately observe the distribution of these cells, they were individually annotated by their biomarkers. For example, T cells were marked by IL7R, CD36 was specifically expressed in the surface of endothelial cells, and fibroblasts were exclusively annotated by COL1A1 and PFGFRA, in addition, CD68, CD14, RGS5 and EPCAM were the markers of macrophages, Pericytes and epithelial cells, respectively (**Figure 3G**). Moreover, the mRNA expression levels of massive protein molecules were observed across these seven cells (**Figure 3H**), revealing that these specifically expressed molecules served as potential biomarkers for corresponding cell types.

Subsequently, the Ucell algorithm was applied for the calculation of the PANoptosis-score among diverse cells as shown in **Figure 3F** as well as it was outlined that this score existed notable distinctions in separate cell types. The mountain map plainly and separately unfolded the divergence of the PANoptosis-score among these seven cell types, finding that this score was distinctive in each cell subtype, and noting that epithelial cells, endothelial cells and fibroblasts possessed higher PANoptosis-score (**Figure 3I**). We further utilized copykat algorithms to identify the tumor cell from epithelial cells in tumor tissues (**Figure 3J**). Corresponding with the results of bulk sequence, in epithelial cells, the PANoptosis-scores of tumor-aneuploid exceeded the score in normal and tumor-diploid samples, suggesting that the extent of PANoptosis was tightly correlated with the development of tumor.

### Deciphering the variations of cell-cell interactions within BC patients

**Figure 4:**
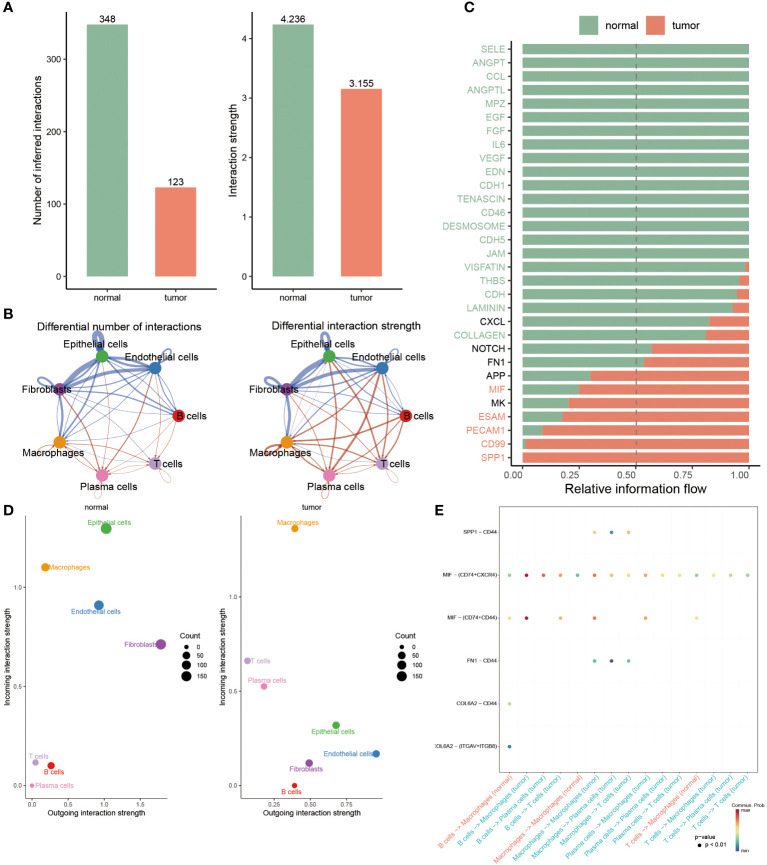
Deciphering the variations of cell-cell interactions within BC patients. **(A)** Comparison of interaction number and strength of multiple cell types between two groups. **(B)** Detailed cell communications among each cell type. **(C)** The bar chart signifies the proportion of massive signaling pathways from diverse cell types in each population. **(D)** Identifying the distinctions of interaction strength of incoming outgoing in separate groups. **(E)** Dot plot revealing the distribution of multiple signaling molecules in B cells, macrophages, T cells and plasma cells between normal and tumor samples.

To clarify the interaction status among these seven cell types (epithelial cells, endothelial cells, B cells, T cells, plasma cells, macrophages, and fibroblasts) in two groups, the bar chart revealed that the numbers and strength of cell-cell interaction in normal groups outperformed BC patients (**Figure 4A**). Subsequently, the network map visually displayed the interaction among seven cells, hence it was found that these three cells, including epithelial cells, fibroblasts and endothelial cells, had stronger interaction in normal populations, accompanied by a weak interaction relationship with the other four cells, such as macrophages, plasma cells, T cells and B cells, in contrast to BC patients (**Figure 4B**). Ulteriorly, the interaction of each intracellular pathway within distinctive groups was identified, witnessing that most of the signaling pathways were notably active in normal populations, such as SELE, ANGPT, CCL, ANGPTL, and other twenty pathways, whereas the activation of seven pathways, namely APP, MIF, MK, ESAM, PECAM1, CD99 and SPP1, primarily occurred in BC patients (**Figure 4C**).

To accurately identify the cell groups in dynamic situations where the received or submitted signals were changed, the comparison based on the outgoing and incoming interaction strength was developed in 4D space. According to this result, it was displayed that epithelial cells, endothelial cells and fibroblasts were classified as primary sources and targets for normal populations, while the chief sources of BC patients were macrophages and plasma cells, indicating the potential that they partook in the progression of BC (**Figure 4D**). Additionally, we further examined the strong interaction probability among T cells, B cells, plasma cells and macrophages. It was seen that the interaction obtained between CDL6A2 and CD44 and ITGAV+ITGB8 exclusively belonged to normal groups, while the interplay between CD44 and SPP1, FN1 merely occurred in BC patients (**Figure 4E**).

### Machine learning approaches to develop a prognostic PANoptosis model

**Figure 5:**
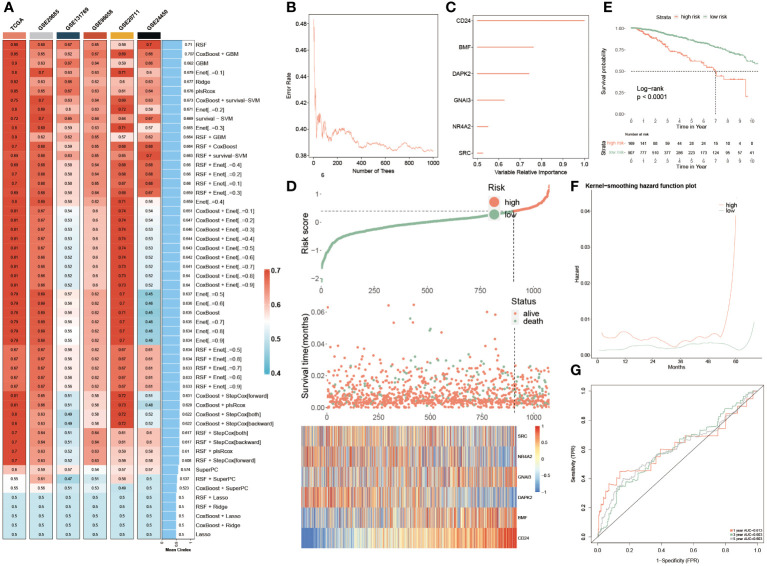
Machine learning approaches to develop a prognostic PANoptosis model. **(A)** The C-indexes of the 55 machine-learning algorithm combinations in the six testing cohorts. (TCGA, GSE20685, GSE131769, GSE96058, GSE2071 and GSE24450). **(B)** The forest model showcases the error rate in several different trees. **(C)** The importance of each significant PANoptosis gene. **(D)** The difference in OS, and survival status between two groups. Heatmap quantifying the expression levels of six PANoptosis genes in distinctive populations. **(E)** KM survival illustrates the survival probability in these two groups. **(F)** The kernel-smoothing hazard function plot demonstrates the correlation between relapse hazard and moths in two populations. **(G)** The ROC curves visualize the AUC values of the PANoptosis-model at one-, three-, and five-year.


In the TCGA dataset and other five testing cohorts, 55 combined algorithms were leveraged to establish the PANoptosis-model, as well as obtained the mean C-index value of each algorithm for each combination (**Figure 5A**). As the result unveiled, the mean C-index of the RSF algorithm was highest compared with other algorithm combinations, emphasizing that this algorithm was competent to recruit prognostic genes and construct a predictive model (**Figure 5A**). To ensure the effectiveness of the subsequent established predictive model, the random forest model was deployed, as well a smaller generalization error (or OOB error) was strongly demanded. It was seen that OOB error was continuously decreasing and dynamic equilibrium between 0.4 and 0.38 (**Figure 5B**). Meanwhile, based on the analysis of random forest, we ultimately recruited six of the most significant genes pertinent to PANoptosis, namely, CD24, BMF, DAPK2, GNAI3, NR4A2 and SRC, which could be utilized to construct a prognostic model further (**Figure 5C**):

$$\begin{array}{l} riskscore=CD24\times 0.3459+BMF\times 0.1461-DAPK2\times 0.1456-\\ GNAI3\times 0.0007-NR4A2\times 0.0413-SRC\times 0.1437 \end{array}$$

Consequently, two subgroups were successfully divided according to multivariate coefficient of the six genes, of which the low-risk group was superior in prognosis, accompanied by longer OS and more alive people, which was in contrast to the high-risk group. Moreover, the expression profiles of these six genes were visualized, of which SRC, NR4A2 and DAPK2 were exceedingly abundant in low-risk populations, while GNAI3, BMF and CD24 exhibited higher expression levels in the high-risk patients (**Figure 5D**). Furthermore, the PANoptosis-model predicted the survival probability of separate-risk patients, displaying that low-risk patients possessed longer OS than high-risk BC patients (**Figure 5E**). The analysis of the Kernel-smoothing hazard also reflected the fact that the high-risk patients possessed a higher probability of BC recurrence compared to low-risk BC patients according to the hazard values (**Figure 5F**). Ultimately, the ROC curve evaluated the predictive ability of this model, since the range of AUC value was between 0.603 and 0.613, demonstrating that it was a robust and reliable prognostic model (**Figure 5G**).

### Predictive performance of the PANoptosis prognostic model

**Figure 6:**
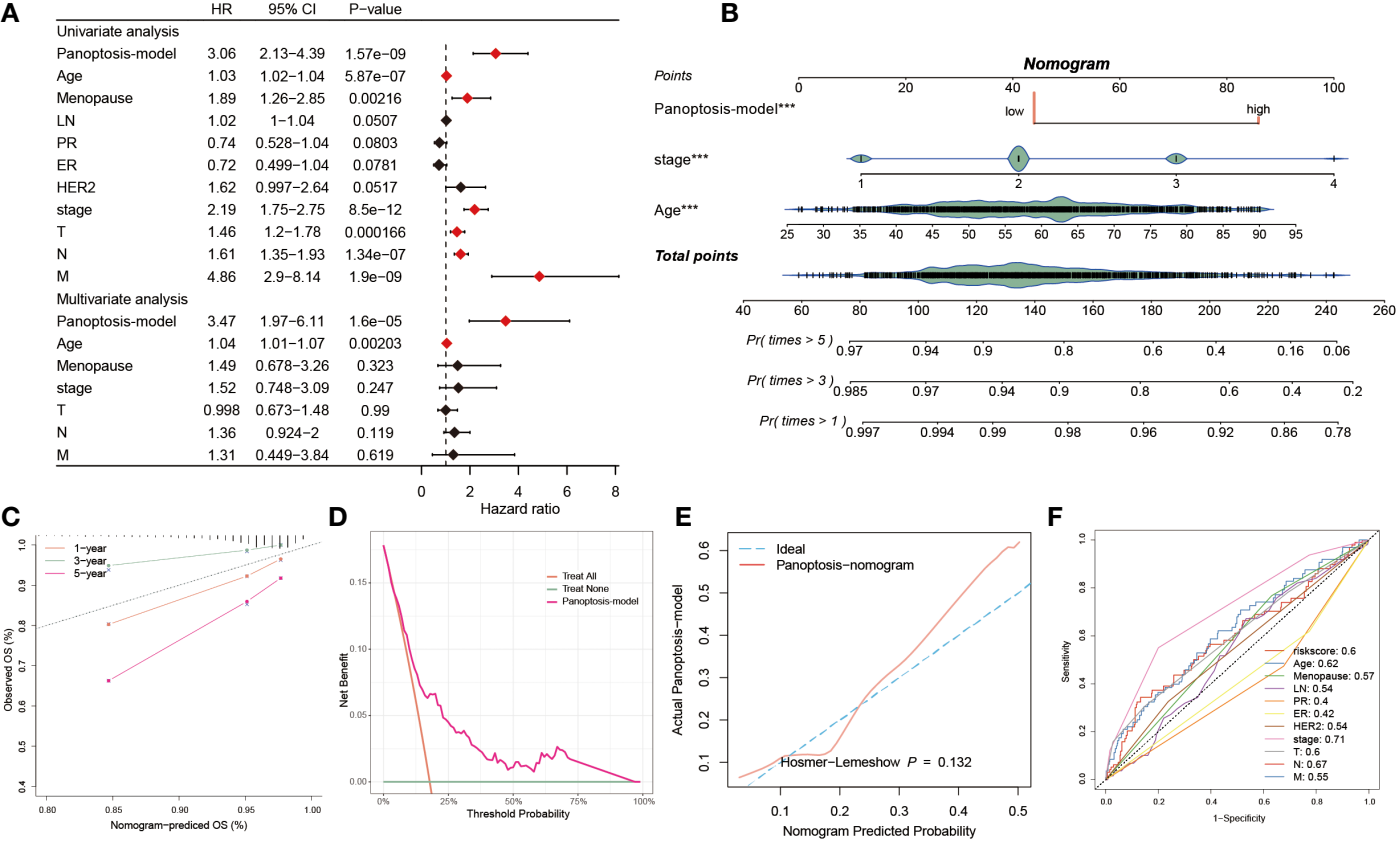
Predictive performance of the PANoptosis prognostic model. **(A)** The univariate Cox regression analysis comprised of risk score, age, menopause, LN, PR, ER, HER2, stage, T, N, and M, and the multivariate Cox regression analysis composed of risk score, age, menopause, stage, T, N, and M. **(B)** The establishment of a PANoptosis-nomogram made up of risk score, stage, and age. **(C)** The calibration curves assess the correctness of this nomogram prediction for OS at 1-, 3-, and 5-year. **(D)** The PANoptosis-model possesses higher net benefit and threshold probability than treat all and treat none. **(E)** The Hosmer-Lemeshow curve of PANoptosis-nomogram plotted by actual PANoptosis-model and nomogram predicted probability. **(F)** 11 ROC curves respectively unfolding the corresponding AUC values of the risk score and ten clinicopathological indexes. ***P < 0.001.

The univariate and multivariate Cox regression analyses were resorted to evaluate the independence of our prognostic model and other clinical factors. The result from univariate Cox analysis indicated that these indicators, including risk score, menopause, stage, T, N and M, were capable of exerting influences on the survival rate of BC patients. Interestingly, the multivariate Cox regression analysis interpreted that risk score and age conformed to the criteria (P< 0.05), demonstrating that our PANoptosis-model was equipped with independence of prediction for BC patients (**Figure 6A**). Since the stage is a valuable reference in clinical practice, we then established a PANoptosis-nomogram to precisely predict the survival probability for BC patients at one-, three-, and five-year, which is composed of risk score, stage and age (**Figure 6B**). As the calibration curves illuminated, the nomogram-predicted OS was extremely consistent with the observed OS, confirming the accuracy of this nomogram (**Figure 6C**). In addition, the decision curve analysis (DCA) and the Hosmer-Lemeshow analysis continued to be conducted to enhance the nomogram persuasiveness. The DCA manifested that the net benefit of the PANoptosis-model curve far outweighed the other two curves, representing the efficiency of this model (**Figure 6D**). Similarly, its salient performance was again validated via the Hosmer-Lemeshow analysis, due to the result that the PANoptosis-nomogram curve did not exist apparent distinction with the ideal curve (P = 0.132) (**Figure 6E**), implying a superb predictive capability of this model. According to these data, it was summarized that the PANoptosis-nomogram had terrific potential and value for clinical application.

We also observed the predictive performance of eleven factors via the ROC curve, of which except for ER (AUC = 0.42) and PR (AUC = 0.4), the AUC value< 0.5, the good predictive potential of other factors was demonstrated (AUC > 0.5, **Figure 6F**). The C-index is a fundamental approach to appraise the predictive capability of distinctive models. We proceeded to assess the predictive efficacy of our model against 100 published signatures through C-index evaluation across the training cohort and ten testing cohorts. Remarkably, our PANoptosis-model demonstrated consistently higher precision compared to other models in the majority of the cohorts, underscoring model’s robustness (**Supplementary Figure S2**).

### Genomic alterations and their prognostic implications in PANoptosis

**Figure 7:**
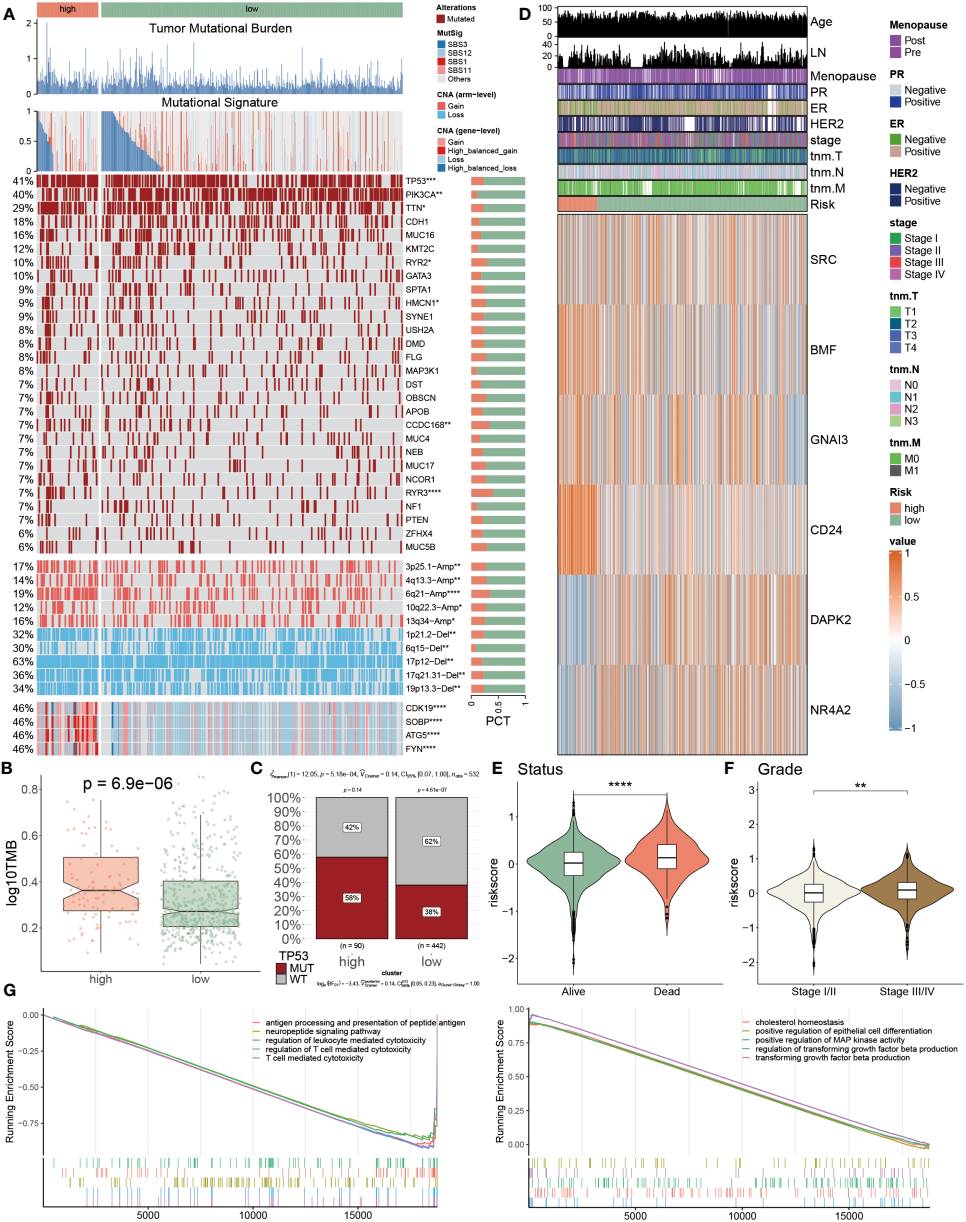
Genomic alterations and their prognostic implications in PANoptosis. **(A)** Genomic alteration landscape according to PANoptosis-model, including TMB, mutational signature, mutated frequencies of top 28 genes, CNV alteration and the distribution of selected genes in Chr6p21; Bar chart visualized the proportion. **(B)** The logarithmic value of TMB was computed in each group. **(C)** The TP53 MUT separately accounted for 58% and 38% in high- and low-risk patients, and similarly the percentages of WT were 42% and 62% between them, respectively **(D)** The heatmap presenting the expression profiling of five important PANoptosis genes in distinct populations and other clinicopathological factors, such as age, LN, stage, and HER2. **(E, F)** The violin charts individually indicated the association of risk score and status **(E)** and grade **(F)**. **(G)** GSEA analyses results from the high-risk subgroup compared with the low-risk one. *P<0.05, **P<0.01, ***P<0.001, ****P < 0.0001.

The above findings had preliminarily mentioned genetic alterations in two groups, to deeply analyze these diversities between two populations, we introduced multi-omics analysis to inspect the genetic variations within separate groups (**Figure 7A**). It was observed that the tumor mutational burden (TMB) of low-risk patients was comparatively lower in comparison to the high-risk populations. Moreover, this finding was again emphasized via the findings from **Figures 7B**, **C**. In **Figure 7B**, we calculated and visualized the TMB value, of which a lower TMB value was detected in low-risk patients instead of in high-risk populations (P< 0.05). Additionally, the TP53 mutational frequency was individually calculated among these two groups, respectively, as well as a higher proportion of the TP53 MUT was discovered in high-risk patients, which accounted for 58% and far surpassed the 38% of low-risk patients (**Figure 7C**). These three mutational signatures of SBS12, SBS1 and SBS11 primarily occurred in high-risk patients. Secondly, it was also detected that the mutation frequency of other genes, such as PIK3A, TTN, HMCN1 and CCDC168, also showed obvious heterogeneity between these two populations. Besides, the amplification of 3p25.1, 6q21 and 13q34, as well as the deletion of 1p21.2, 17p12, 17q21.31 and 19p13.3 were identified in high-risk patients. These results were demonstrated due to the gain of oncogenic genes CDK19, SOBP, ATG5 and FYN in chr6q21 (**Figure 7A**). After that, the expression levels of six PANoptosis-related genes were quantified, as the heatmap showcased that SRC, DAPK2 and NR4A2 were primarily up-regulated in low-risk BC patients, while other three PANoptosis genes had higher expression levels in other risk patients (**Figure 7D**). Subsequently, the relevance between the risk score and survival status (**Figure 7E**) and tumor grade (**Figure 7F**) was explained, respectively. The risk score presented a remarkably positive correlation with status and grade, hinting that PANoptosis was pivotal in BC development. Ultimately, we compared the enrichment divergences of landmark pathways between two populations using GSEA analysis, of which five signaling pathways related to the immunoreaction had lower abundances in the high-risk patients, such as T cell-mediated cytotoxicity, antigen processing and presentation of peptide antigen, taking the positive regulation of epithelial cell differentiation pathway as an example, these five pathways, which were involved in cell development, were dramatically down-regulated in the low-risk patients (**Figure 7G**).

### Tumor microenvironment evaluations using the PANoptosis model

**Figure 8:**
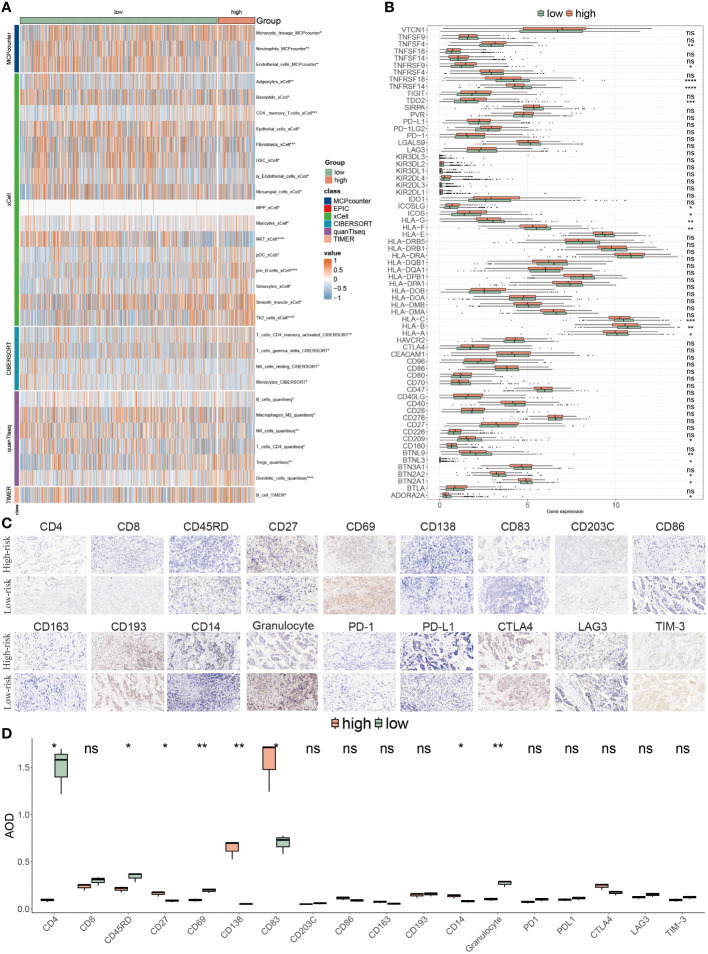
Tumor microenvironment evaluations using the PANoptosis model. **(A)** The distribution of multiple infiltrated immune cells derived from five algorithms (xCell, CIBERSORT, quanTIseq, TIMER) in two populations. **(B)** The expression features of massive immune checkpoints in these two risk-score subgroups. **(C)** IHC images of infiltrated immune cells and ICIs targeting the representative makers. **(D)** Statistical result of **(C)**. *P<0.05, **P<0.01, ***P<0.001, ****P < 0.0001. ns, not significant.

Due to the significance of the TME in tumor development, we explored the immune discrepancies between these two populations using multiple methods, including CIBERSORT, xCell, MCP counter, TIMER and quanTIseq, to deeply analyze the prognostic mechanisms. We observed that a majority of immune cell types were prominently distributed in low-risk BC patients, such as neutrophil cells, monocyte cells, CD4^+^T cells and NK cells, and a minority of cells were found in the high PANoptosis-score patients, for instance, pDC cells, Tregs, and dendritic cells (**Figure 8A**). Consistent with immune infiltration, low-risk patients possessed a higher abundance of ICIs compared to high-risk patients, accompanied by a favorable prognosis (**Figure 8B**). To further assess the TME variation and validate the analyzed results, we performed IHC staining based on key cell markers and ICIs in-house collected samples, the representative IHC images and statical results are shown in **Figures 8C, D**.

### Prognostic implications of PANoptosis for ICIs therapy response

**Figure 9:**
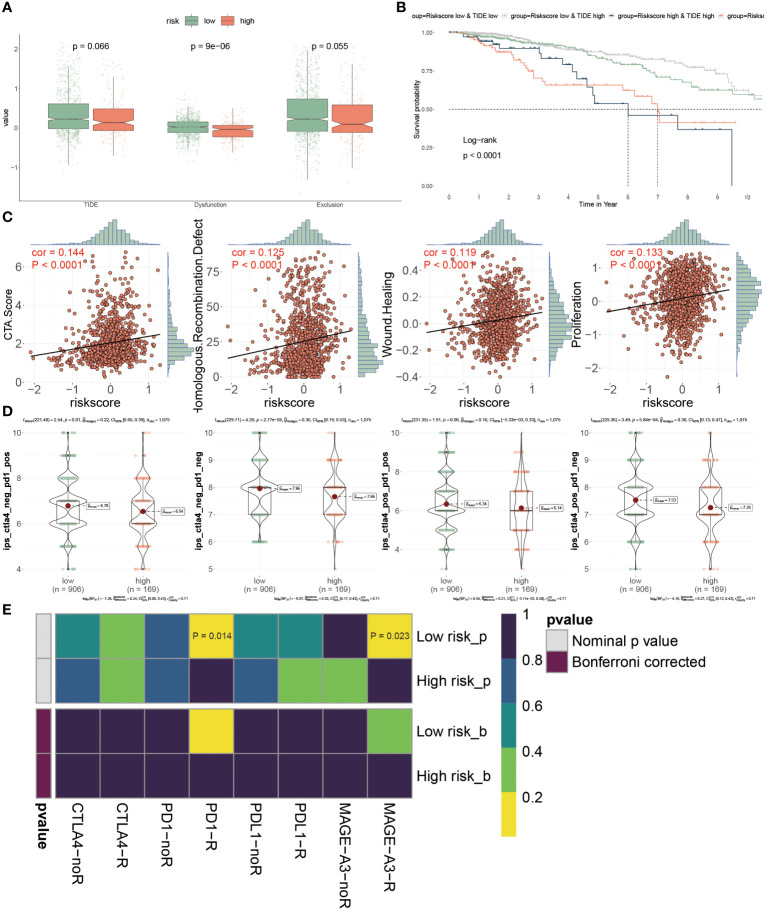
Prognostic implications of PANoptosis for icis therapy response. **(A)** Differences of TIDE value, dysfunction value and exclusion value in low- and high- risk populations. **(B)** To compare the OS of four combinations composed of (low or high) TIDE and (low or high) risk score, individually. **(C)** The relevance of the risk score and CTA score, recombination defect, wound healing and proliferation. **(D)** IPS (Immunophenoscore) value of each combination among two risk groups. **(E)** Putative ICIs therapy response in two risk BC patients.

Through the evaluation of TME, it was speculated that low-risk BC patients may be superior in response to immunotherapy based on more immune cell infiltration and higher expression levels of ICIs genes, consequently demanding further validation. It is well-known that TIDE has been widely utilized to examine the efficiency of immunotherapy, and it typified a negative relevance with the responsiveness. Here, higher Dysfunction values were observed in low-risk patients, but there were no prominent differences in the TIDE and Exclusion scores between these two patients (P > 0.05) (**Figure 9A**). The survival probabilities included four combinations that were separately evaluated as shown by four KM survival curves, suggesting that low-risk score and high TIDE were superior in the outcome than other combinations, which claimed that low-risk patients with high TIDE value implied the further improvement of prognosis effect and risk score played a domain and decisive role (**Figure 9B**).

We estimated the tumor immunogenicity characteristics according to these indexes including CTA score, recombination defect, wound healing and proliferation, since their dysregulation could furnish new impetus for the development of tumors. As the outcomes exhibited, these four indexes displayed sensibly positive relations with the risk score, proposing that high-risk BC patients possessed inferior prognoses (**Figure 9C**). Until now, it has not been determined which groups were more suitable for ICIs therapy, so we introduced the IPS score from the TCGA dataset to diagnose. It was emphasized that the IPS scores were all extremely high in low-risk BC patients, representing that this group was more likely to acquire benefits from immunotherapy, no matter what alone or combined therapy methods were (**Figure 9D**). Ultimately, the assessment of response to PD1, PDL1, CTLA4 and MAGE-A3 treatment provided the immunotherapy chance for high-risk populations, but this group was merely limited to response to anti-PD-1 and MAGE-A3 treatments (P< 0.05) (**Figure 9E**). Collectively, the PANoptosis-model predicted the responsiveness to ICIs therapy between distinctive groups, as well as low-risk BC patients were more beneficial for this treatment in clinical.

### Chemotherapy response and PANoptosis signatures

**Figure 10:**
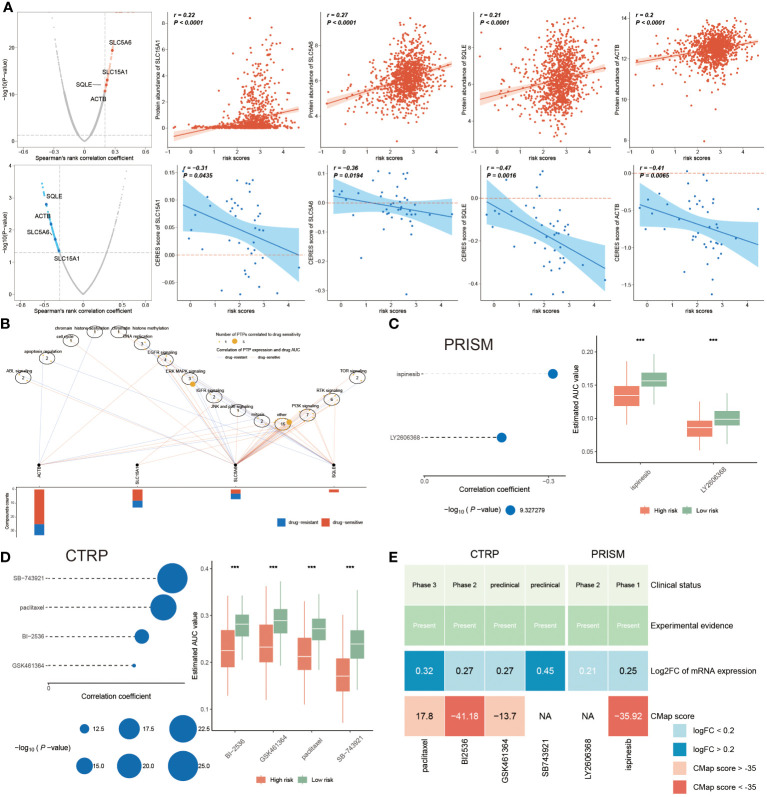
Chemotherapy response and PANoptosis signatures. **(A)** The left plots illuminating the spearman’s rank correlation coefficient of five candidates, among them the red and blue plots signifying the positive and negative correlations, respectively. Correspondingly, the right scatter plots separately denoting the relevance between the risk score and each candidate’ protein abundance and CERES score. **(B)** Spearman correlation between mRNA expression of potential targets and drug sensitivity across cancer cell line. **(C, D)** Correlation coefficients of two compounds from PRISM dataset **(C)** and of four compounds gotten from CTRP **(D)**, of which the larger circle, the lower *P*-value. Accordingly, the right boxplot illustrating the remarkable distinction of AUC value between these two risk groups in each compound. **(E)** A multiple-perspective analysis was constituted by clinical status, experimental evidence, mRNA expression and CMap score of six compounds. ***P < 0.001.

While novel therapeutic approaches, including targeted therapy, have been explored, chemotherapy remains an essential option for clinical cancer treatment. Therefore, it is imperative to employ the PANoptosis model to predict chemotherapy response in BC patients, to improve the prognosis, especially for high-risk BC patients. The identification of therapeutic targets is deemed a pivotal measurement to breaking undruggable dilemmas. So, Spearman’s correlation analysis was utilized to select, finding that four proteins had a higher abundance in high-risk patients, which also indicated that this group was more prone to chemotherapy. Moreover, the CERES scores also supported the finding that these four proteins were considered therapeutic targets for high-risk BC patients (**Figure 10A**). Meanwhile, the candidate drug targeting these four anti-cancer drugs was characterized as having higher drug sensitivity (**Figure 10B**). Together, ACTB, SLC15A1, SLC5A6, and SQLE were recommended as potential therapeutic targets.

In the following, we continued to identify underlying drugs from PRISM and CTRP datasets. This study displayed that a total of six candidate compounds were listed, among them ispinesib and LY2606368 compounds from the PRISM dataset (**Figure 10C**), and the CTRP dataset including SB-743921, paclitaxel, BI-2536 and GSK461364 compounds (**Figure 10D**). We also found that lower AUC value was reported in high-risk patients, hinting a better responsiveness to chemotherapy in this population. Since the above finding was unable to determine the most superior drugs, therefore, a multiple-perspective analysis was further conducted. Among them, the clinical status, experimental evidence, mRNA expression levels and CMap score of these six compounds were disclosed, based on the criterion CMap score< -35, concluding that only BI-2536 and ispinesib were ultimately chosen as therapeutic drugs for high-risk BC populations (**Figure 10E**; **Supplementary Table S6**).

## Discussion

Our investigation into the role of PANoptosis in breast cancer marks a significant stride toward refining prognostic tools and personalizing patient care. By developing a risk score rooted in the intricate mechanisms of PANoptosis—encompassing pyroptosis, apoptosis, and necroptosis—we’ve unveiled a multifaceted perspective on tumor biology and patient outcomes. This approach not only sheds light on the underlying processes driving breast cancer progression but also sets the stage for targeted therapeutic strategies that could dramatically alter the clinical landscape.

The introduction of our PANoptosis-based risk score has the potential to revolutionize treatment paradigms in breast cancer care. By accurately stratifying patients according to their risk, we can pave the way for more nuanced treatment strategies—those at higher risk might benefit from innovative, aggressive treatments earlier in their disease course, while lower-risk patients could avoid unnecessary side effects from overtreatment. Moreover, our findings underscore the potential of targeting the PANoptosis pathway as a novel therapeutic avenue, offering hope for treatments that could inhibit tumor growth and metastasis more effectively.

Crucially, our study illuminates the complex relationship between PANoptosis and key processes in cancer development. While our analysis provides valuable insights into PANoptosis and its association with breast cancer prognosis, we did not directly investigate its relationship with EMT or other specific pathways involved in cancer metastasis. However, the identification of key PANoptosis-related genes and their prognostic significance underscores the potential of further research in these areas. Future studies could explore how these genes interact within the broader network of cancer progression mechanisms, potentially uncovering novel targets for therapeutic intervention. Understanding how PANoptosis intersects with other cellular processes to drive or inhibit cancer spread is essential for developing targeted interventions that could halt progression and improve survival outcomes.

While our study represents a pivotal step forward, we must acknowledge the limitations inherent in our current model, most notably the exclusion of molecular subtypes of breast cancer. This oversight is significant; the diversity of breast cancer at the molecular level profoundly influences both prognosis and therapeutic response. Future iterations of our research will need to incorporate these subtypes to fully capture the complexity of breast cancer and refine the applicability of our risk score in diverse clinical contexts.

Looking forward, integrating our PANoptosis-based risk score with molecular subtyping and other biomarkers could yield a robust, multifactorial tool for breast cancer prognosis and treatment planning. Collaborative research efforts that bridge basic science and clinical practice are essential for translating these insights into tangible benefits for patients. Moreover, exploring the potential of combination therapies that target both PANoptosis pathways and other key drivers of tumor growth and resistance may offer new hope for challenging cases of breast cancer.

In sum, our study contributes a vital piece to the puzzle of breast cancer prognosis and treatment, highlighting the importance of PANoptosis in shaping patient outcomes. While challenges remain in translating these findings into clinical practice, the promise of more personalized, effective treatment strategies based on our understanding of PANoptosis offers a new horizon in breast cancer care. As we move forward, expanding the scope of our research to address the limitations identified and exploring the full therapeutic potential of targeting PANoptosis will be crucial in our ongoing battle against breast cancer.

## Data availability statement

The datasets presented in this study can be found in online repositories. The names of the repository/repositories and accession number(s) can be found in the article/**Supplementary Material**.

## Ethics statement

The studies involving humans were approved by Ethics Committee of Guizhou Provincial People’s Hospital (approved number: 2023-070). The studies were conducted in accordance with the local legislation and institutional requirements. The participants provided their written informed consent to participate in this study.

## Author contributions

SW: Data curation, Formal analysis, Writing – original draft. ZL: Investigation, Resources, Visualization, Writing – original draft. JH: Methodology, Resources, Writing – original draft. XL: Investigation, Resources, Writing – original draft. QN: Funding acquisition, Methodology, Supervision, Writing – review & editing. TW: Conceptualization, Formal analysis, Funding acquisition, Visualization, Writing – original draft, Writing – review & editing.
