# Supplementary figures and images for: Integrating PANoptosis insights to enhance breast cancer prognosis and therapeutic decision-making
Source: Front Immunol. 2024 Mar 5;15:1359204. doi: 10.3389/fimmu.2024.1359204 (PMC10948567; doi:10.3389/fimmu.2024.1359204)

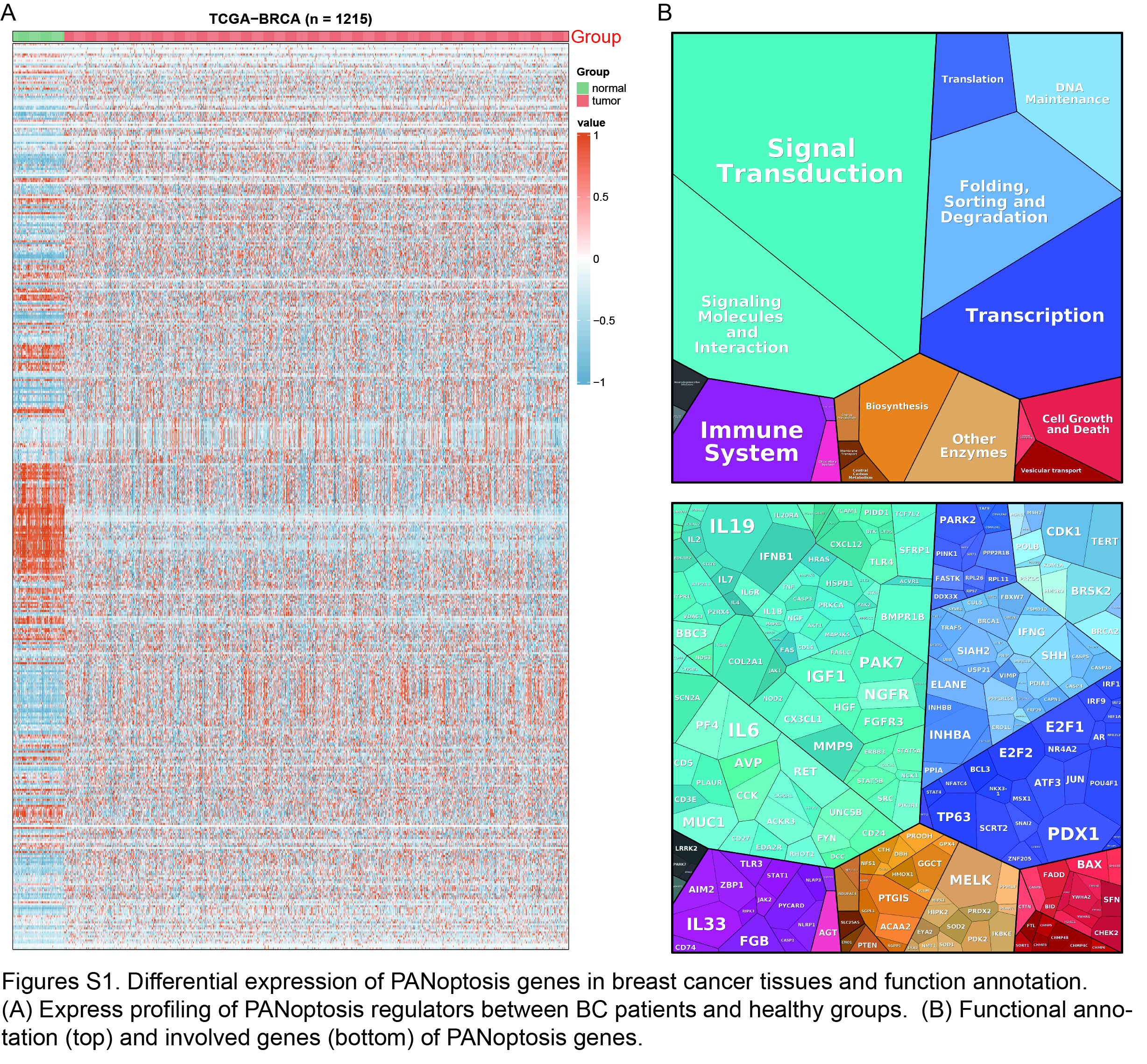

Supplement: Supplementary file 7 [file Image_1.tif]

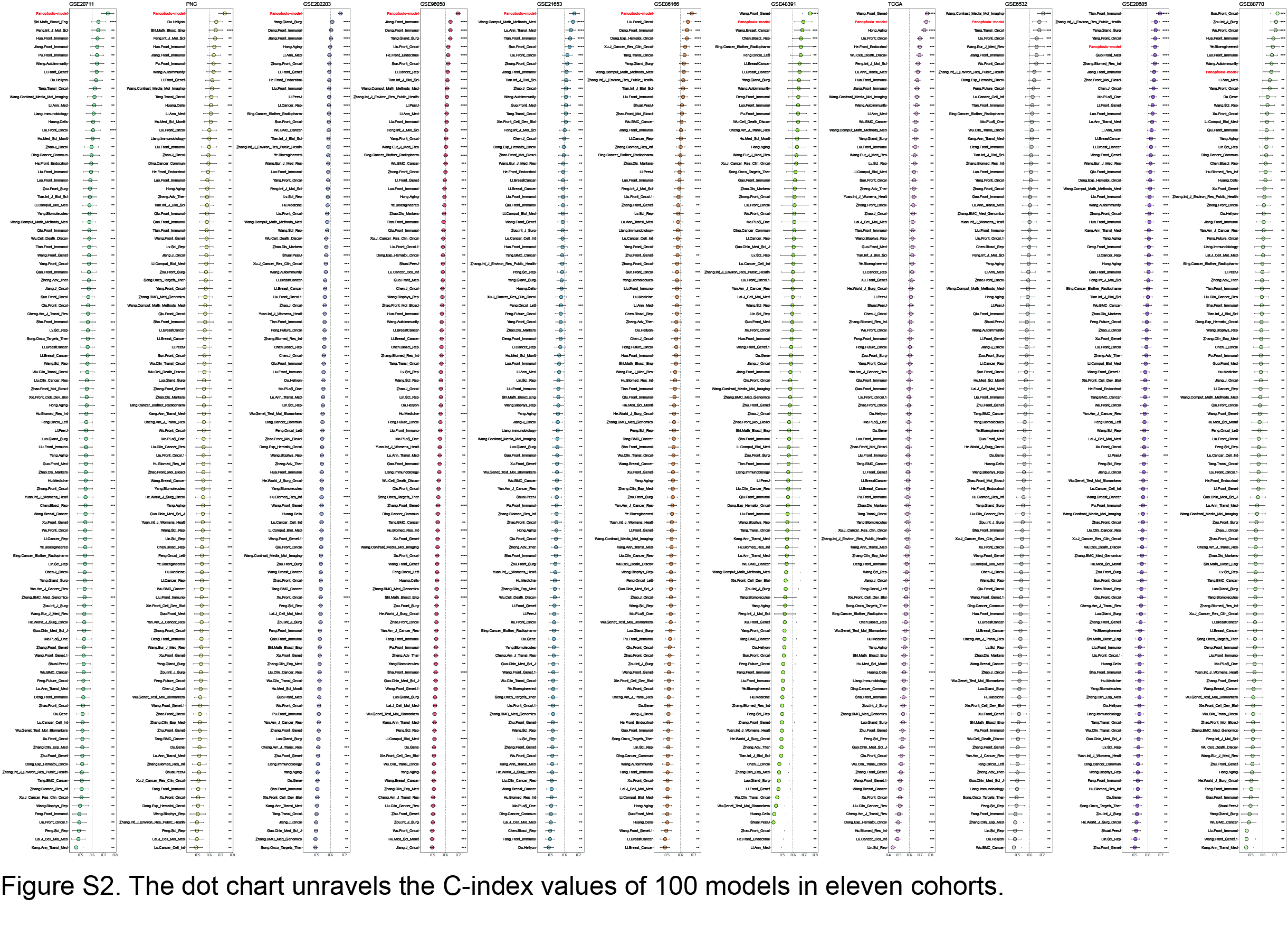

Supplement: Supplementary file 8 [file Image_2.tif]
